# Repeated intravenous thrombolysis in early recurrent stroke due to free-floating thrombus

**DOI:** 10.1055/s-0044-1792090

**Published:** 2025-01-15

**Authors:** Luis Eduardo Borges de Macedo Zubko, Felipe Trevisan Matos Novak, Marcos C. Lange

**Affiliations:** 1 Universidade Federal do Paraná, Complexo Hospital de Clínicas, Divisão de Neurologia, Curitiba PR, Brazil.

**Figure 1:**
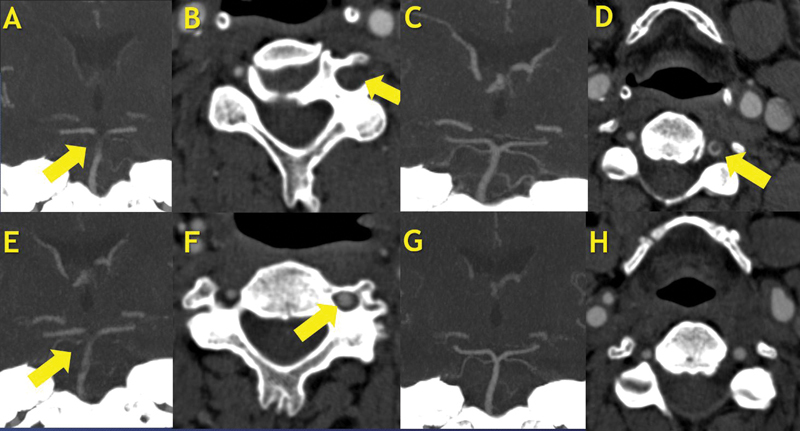
( **A–B** ) Admission computed tomography angiography (CTA) showing occlusion of the basilar artery and left vertebral artery. ( **C–D** ) Computed tomography angiography, taken on day 2, after the first thrombolysis, showing basilar artery recanalization and a free-floating thrombus in the left vertebral artery. ( **E–F** ) Computed tomography angiography, taken on day 3, showing dislocation of the left vertebral artery to the basilar artery causing a new stroke. ( **G–H** ) Complete recanalization of the basilar and vertebral artery after the second thrombolysis.

A 52-year-old man presented with acute vertigo, bilateral myoclonic-convulsive movements, anisocoria, upbeat nystagmus, skew deviation, quadriparesis, and right-sided dysmetria. His initial score on the National Institutes of Health Stroke Scale (NIHSS) upon hospital admission was of 22. A head computed tomography angiography (CTA) scan showed occlusion of the basilar artery and left vertebral artery ( Figure 1 A–B ). He was treated with alteplase, with onset-to-treatment time of 2 hours and 29 minutes, resulting in complete and early neurological improvement (NIHSS score of 0). On day 3, he presented acute altered mental status associated with quadriparesis, myoclonic-convulsive movements, dysphagia, and sialorrhea (NIHSS score of 20). Another CTA scan showed a new basilar artery occlusion, due to dislocation of the left vertebral artery free-floating thrombus, measuring 59 mm in craniocaudal length 1 ( Figure 1 C–H ). The patient was once again treated with alteplase, due to unavailability of mechanical thrombectomy at our institution at the time, with onset-to-treatment time of 17 minutes, leading to complete neurological improvement (score of 0 on the NIHSS and on the Modified Rankin Scale [mRS]). This case highlights the importance of repeating vessel studies, and it shows that repeat intravenous thrombolysis may be an option for patients with early posterior circulation recurrent strokes, whereas it may carry a higher risk of intracranial hemorrhage in anterior circulation large vessel occlusion strokes. 2 3
